# Internal biliary drainage superior to external biliary drainage in improving gut mucosa barrier because of goblet cells and mucin-2 up-regulation

**DOI:** 10.1042/BSR20171241

**Published:** 2018-05-28

**Authors:** Xuechan Tang, Weiping Ma, Weirong Zhan, Xin Wang, Huan Dong, Hongjing Zhao, Lin Yang, Cuiying Ji, Qing Han, Chenguang Ji, Hongqun Liu, Na Wang

**Affiliations:** 1 Department of Gastroenterology, the Second Hospital of Hebei Medical University, Hebei Key Laboratory of Gastroenterology, Hebei Institute of Gastroenterology, 215 Heping West Road, Xinhua District, Shijiazhuang 050000, Hebei Province, China; 2 Department of Gastroenterology, Aviation General Hospital of China Medical University & Beijing Institute of Translational Medicine, Chinese Academy of Sciences, No.3 Anwai beiyuan Road, Chaoyang District, 100012, Beijing, China; 3 Department of Gastroenterology, the Han Dan Central Hospital, 59 Congtai North Road, Gaokai District, Handan 056000, Hebei Province, China; 4 Department of Laboratory Medicine, Department of Biotechnology, School of Medical Science, School of Applied Science, Royal Melbourne Institute of Technology University, Plenty Road, Bundoora VIC 3083, Australia

**Keywords:** External biliary drainage, Goblet cell, Internal biliary drainage, Intestinal mucosal barrier, Mucin-2, Obstructive jaundice

## Abstract

Backgroud: Obstructive jaundice increases intestinal permeability, but the pathological mechanisms remain obscure, which results in debates about the necessity of performing preoperative biliary drainage in patients with obstructive jaundice. Mucin-2 (MUC2) and goblet cells regulated by bile acids play an important role in maintaining the function of intestinal mucosal barrier. The present study was to investigate the role of goblet cells and MUC2 in obstructive jaundice and evaluate the effect of biliary drainage on intestinal permeability.

Study design: We enrolled patients with malignant biliary obstruction and controls. We also did animal studies with four groups of rats: sham operation, obstructive jaundice, internal biliary drainage, and external biliary drainage. Histopathological analysis, biochemical measurement, and electron microscopy examination were done on pertinent samples.

Results: Compared with the control group, the small intestinal mucosa was significantly damaged; goblet cells and MUC2 were significantly decreased and serum endotoxin level was significantly increased in patients and rats with obstructive jaundice. Biliary drainage, especially internal biliary drainage, significantly increased goblet cells and MUC2 and attenuated the damage of small intestinal mucosa.

Conclusions: In obstructive jaundice condition, goblet cells and MUC2 were reduced which were involved in the damage of intestinal mucosa barrier; biliary drainage increased goblet cells and MUC2, repaired mucosa layer and restored the intestinal mucosa barrier function.

## Introduction

Various pathological conditions such as extrahepatic and intrahepatic biliary obstruction induce obstructive jaundice and associated complications. Damaged intestinal mucosal barrier due to obstructive jaundice results in the postoperative morbidity and mortality in patients [1]. However, the necessity of preoperative biliary drainage (PBD) is still in debate [2–5]. One perspective is that the procedure causes more complications and therefore, the PBD should be evaluated based on patients’ medical conditions [2,3]. On the contrary, portal and systemic endotoxemia because of unbalanced bacteria in the intestinal tract is a proinflammatory factor. The animal studies show that PBD is an important procedure to reduce the fatal inflammation [4,5]. In addition, different procedure types including internal biliary drainage and external biliary drainage are discussed in different studies [6–9]. Some studies are in favor of internal biliary drainage in recovery of enterohepatic circulation, improvement of intestinal barrier and reduction of endotoxin [6,7], others indicate that the risk of certain complications such as cholangitis associated drainage tube occlusion is increased by internal biliary drainage compared with external biliary drainage [8,9].

The intestinal epithelium is formed by enterocyte, paneth cell and goblet cell, which are the central components of the intestinal mucosal barrier [10]. Mucins, particularly mucin-2 (MUC2), secreted from goblet cells, prevent pathogenic microorganism colonization, translocation and interaction of enterotoxin to the internal milieu from bacteria [11]. Animal studies demonstrated that MUC2-deficient mice have chronic colonic inflammation [12], and dextran sulfate sodium-induced chronic intestinal inflammation has mucus barrier damage in mice colon [13]. MUC2 contains apical granules consistently in response to certain external stimuli [10,14]. Meanwhile, accelerated secretion of MUC2 due to exocytosis of goblet cells can cause acute release of centrally stored mucin granules [14,15]. In addition, the expression of MUC2 regulated by bile acids (deoxycholic acid) is a dose- and time-dependence in human esophagus [16]. The number of goblet cells is pH related. Theodorou and colleagues found that the growth and differentiation of goblet cells are positively related to the pH within the range from 2.2 to 4.4 in human Barrett’s esophagus [17]. As a result, the change of gut intraluminal pH associated with volume of bile acids might regulate the amount of goblet cells.

Due to the situation that the mechanism of breakage of intestinal mucosal barrier is not fully understood, we hypothesized that the decreased expression of MUC2 in small intestine because of the absence of bile acids might be one of the mechanisms causing intestinal mucosa barrier damage in patients with obstructive jaundice. We also hypothesized that internal biliary drainage is more effective to relieve the clinical manifestation via increasing the number of goblet cells.

## Methods

### Clinical study

The present study was approved by the Ethics Committee of The Second Hospital, Hebei Medical University. Clinical participants were informed and consent forms were signed. Twenty-two out of 32 patients with malignant biliary obstruction were divided into two groups: malignant obstructive jaundice group (serum bilirubine ≥ 43 mM, *n*=14) and malignant biliary obstruction without jaundice (*n*=8). They were examined by endoscopic retrograde cholangiopancreatography. Results from CT and ultrasound scanners revealed no evidence of metastases in all of the patients. Patients with negative gastroscopic examination and normal liver function test were assigned as controls. The characteristics of participates are referred to Table 1.

**Table 1:** Characteristics of the patient participates in three different groups

Characteristics	MOJ group	NJ group	CON group
**Patients**	14	8	10
**Sex (male/female)**	9/5	5/3	6/4
**Age (years)**	68.86 ± 10.205	62.25 ± 10.55	66.5 ± 11.16
**TBIL (μmol/l)**	206.26 ± 110.06*	14.48 ± 4.04	11.7 ± 3.96
**ALP (U/l)**	546.4 ± 416.6	135.26 ± 148.52	67.29 ± 10.34
**γ-GT (U/l)**	738.9 ± 357.89	168.37 ± 270.07	17.0 ± 4.89
**ALT (U/l)**	212.67 ± 145.2	59.51 ± 107.37	21.5 ± 10.3
**AST (U/l)**	198.6 ± 95.29	52.22 ± 77.92	22.7 ± 5.40
**Diagnosis**	periampullary	periampullary	normal
	carcinoma (*n*=9)	carcinoma (*n*=6)	(*n*=10)
	cholangiocarcinoma (*n*=5)	cholangiocarcinoma (*n*=2)	

Note:*P<0.05 vs NJ group and CON group.

Abbreviations: γ-GT, γ-glutamyltransferase; ALP, alkaline phosphatase; ALT, alanine aminotransferase; AST, aspartate aminotransferase; CON, control; MOJ, malignant obstructive jaundice; NJ, nonjaundice; TBIL, total bilirubin.

**Table 2:** The biochemical test results of TBIL, ALT, and AST across different groups

Group (*n*)	TBIL (μmol/l)	ALT (U/l)	AST (U/l)
SH (*n*=20)	21.46 ± 3.45	40.79 ± 9.24	88.72 ± 29.31
OJ (*n*=14)	107.72 ± 18.22*	122.82 ± 36.23*	409.48 ± 73.06*
ID (*n*=18)	30.51 ± 12.44†	56.79 ± 19.66^†^	178.49 ± 67.52^†^
ED (*n*=16)	35.89 ± 15.57^†^	59.44 ± 20.11^†^	109.09 ± 38.06^b‡^

Note: Biochemical tests compared with biochemical tests in SH group, ^*^P<0.01. Biochemical tests compared with biochemical tests in OJ group, ^†^*P*<0.01. Biochemical tests compared with biochemical tests in ID group, ^‡^*P*<0.05.

Abbreviations: ALT, alanine aminotransferase; AST, aspartate aminotransferase; ED, external biliary drainage; ID, internal biliary drainage; OJ, obstructive jaundice; SH, sham operation; TBIL, total bilirubin.

### Animal studies

The animal studies were approved by the Animal Ethics Committee of Hebei Medical University (Shijiazhuang, China). A total of 80 male Sprague–Dawley rats (8 weeks old, 200–250 g) were included. All of the rats were housed under the constant condition of temperature (21 ± 2°C) and humidity with 12-h dark/light cycles. In addition, rats were given *ad libitum* access to pellets and tap water.

#### Animal experiment procedures design

**Figure 1:**
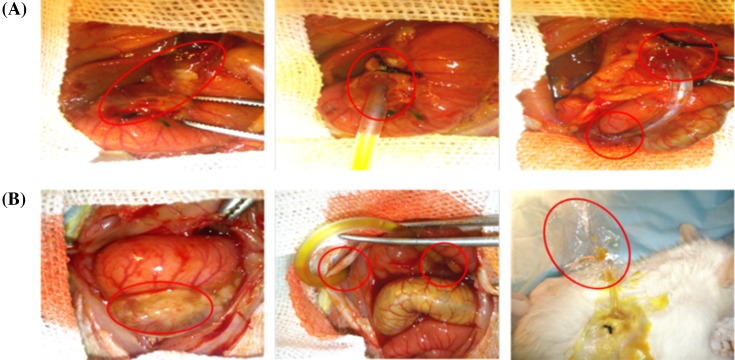
Bile duct ligation rats mimic preoperative biliary drainage (PBD) in patients (**A**) The procedure of internal biliary drainage. (**B**) The procedure of external biliary drainage.

Rats were randomly allocated into four groups (10 per group). Group 1: sham operation; Group 2: obstructive jaundice (common bile duct ligation, BDL); Group 3: BDL + internal biliary drainage (Figure 1A), and Group 4: Group BDL + external biliary drainage (Figure 1B). All of the procedures were performed under sterile condition and rats were anesthetized with 2% sodium pentobarbital (30 mg/kg). After midline incision, the common bile duct was separated and double ligated with 3-0 silk. Sham-operation was the same but no bile duct ligation. The abdominal wall was closed with 2-0 silk continuous sutures. The second laparotomy was performed after 7 days. Rats’ duodenums from Group 1 and Group 2 were dissected without other treatments. However, the implantation of a drainage tube between the dilated common bile duct end and duodenum was set up in Group 3 rats, and exteriorization with a drainage tube at the nape was set up in Group 4 rats. Blood samples and ileum segments were collected at day 7 of second post laparotomy.

#### Biochemistry

Rat serum specimens were collected to measure total bilirubin (TBIL), aspartate aminotransferase (AST), and alanine aminotransferase (ALT) by autoanalyzer (Abbott Ci8200 system, Abbott Laboratories, Abbott Park, IL). The serum endotoxin levels were measured according to the manufacturer’s instructions (Horseshoe Crab Reagent, Chenjiang, China).

### Histopathological analysis

#### Tissue collection and processing

In clinical study, biopsy specimens of the second part of the duodenum, distal to the ampulla of Vater were obtained from all participants. In animal study, the segments of ileum were collected during the laparotomy. Dissected segments of small intestine were fixed, embedded, and sectioned with 5-µm thickness.

#### Hematoxylin–eosin and special staining

Sections were hematoxylin–eosin (H&E) stained for small intestine damage evaluation. Mucins in the duodenum were stained by Alcian Blue–periodic acid Schiff (AB–PAS) in order to examine the density of goblet cells in intestine tissues from both human and rats and the mucus layer thickness of rats’ ileum was measured. Both transversal and U-shaped longitudinal sections were displayed and stained for this research. The number of goblet cells and the thickness of mucus layer of ileum from each clinical and animal samples were assessed by Image-Pro Plus 6.0 view software (Media Cybernetics, America).

#### Immunohistochemistry

Before staining, antibody was optimized and antigen was retrieved. The sections were deparaffinized and rehydrated. With the condition of citrate buffer pH 6 pretreatment, monoclonal primary antibody against MUC2 (EPR6145, ab134119, Abcam diluted 1:1000) was applied for the immunohistochemical staining. After phosphate-buffered saline (PBS) washing, sections were incubated with secondary antibody coupled to EnVision/HRP (zhongshan goldenbridge biotechnology CO). All of the results were visualized by Image-Pro Plus 6.0 view software (Media Cybernetics, America).

### Electron microscopy

Tissues were immersed in 3% glutaraldehyde fixative buffered in 0.09 M KH_2_PO_4_ at pH 7.4. Samples were then washed in 0.09 M KH_2_PO_4_ buffer with 7.5% sucrose and transferred to a 1% OsO_4_ + 1.5% ferrocyanide solution and buffered to pH 7.4 with 0.1 M veronal-acetate for subsequent postfixation for 1 h at 48 °C. After washing in phosphate veronal-acetate buffer containing 7% sucrose at pH 7.4, dehydration was carried out rapidly in graded ethanol series followed by embedding in Epon. Tissue sections were cut and examined with a Hitachi H-7500 electron microscope.

### Statistical analysis

Statistical analysis was performed using SPSS 18 for Windows (SPSS, Chicago, Illinois, U.S.A.). One-way analysis of variance (ANOVA) was used for the comparison of control, malignant biliary obstruction with or without jaundice groups, and the SNK-q test was followed. All data are presented as mean ± SD. A *P* value <0.05 was considered statistically significant.

## Results

### Human specimen evaluation

#### Hematoxylin–eosin staining

**Figure 2:**
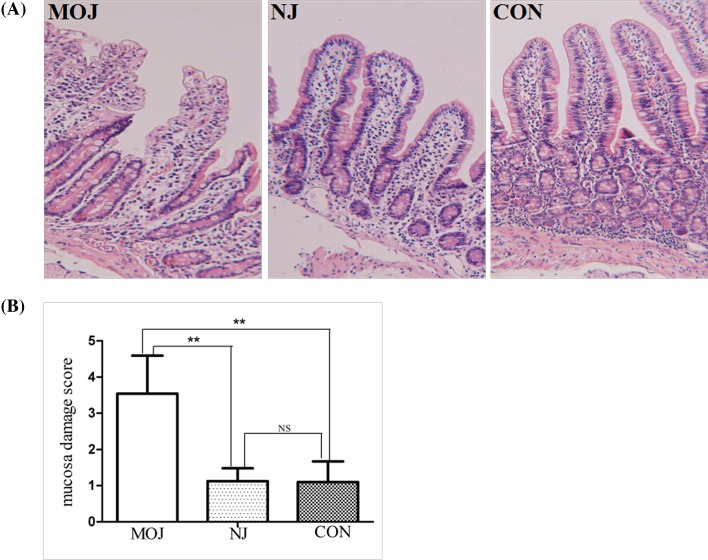
Intestinal mucosal injury of patients in different groups (**A**) H&E staining. (**B**) intestinal mucosal injury according to Chiu’s score. The score was significantly higher in MOJ group compared with other two groups (**P<0.01). There is no difference between NJ and CON groups; CON, control; MOJ, malignant obstructive jaundice; NJ, nonjaundice; NS, not significant.

Microscopic description of human sections from endoscopic duodenal biopsies with H&E staining indicated irregular shape transverse section with major eosinophilic appearance. Microscopic description indicated different images from different groups and natural edge with basophilic nucleus and eosinophilic cytoplasm cell presentation. The majority presentation of the tissue is four layers including mucosa, submucosa, muscularix externa and seroa, and particularly the intestinal villi demonstrated various degrees of short, thick and edematous appearance across all groups. However, intestinal villi from malignant obstructive jaundice group (Figure 2A) presented significantly impaired integrity, and there were enlarged interior margin between the lamina propria and epithelium at the tops of the villi with clear epithelial necrosis. In addition, interior margins were found on the two sides of the villi with partly damage of the epithelium. On the other side, microscopic description of nonjaundice group sections (Figure 2A) and control group sections (Figure 2A) illustrated normal conditions of finger-like protrusions villi covered by simple columnar epithelium, and no inflammatory cell infiltration was observed. Furthermore, we evaluated the severity of intestinal mucosal injury according to Chiu’s scores [18], indices of intestinal mucosal injury, under the light microscope by randomly counting in ten fields of each sample. The score of malignant obstructive jaundice group (3.54 ± 1.05) was higher than the score of nonjaundice group (1.13 ± 0.35) and control group (1.10 ± 0.57), which indicated statistical significance (Figure 2B, *P*<0.01).

#### AB–PAS *staining*

**Figure 3:**
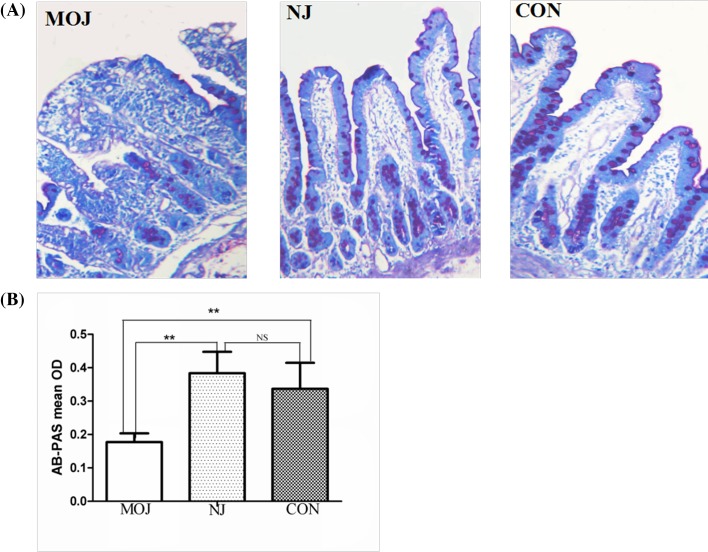
Goblet cell contents in different groups (**A**) AB–PAS staining. (**B**) The mean optical density of goblet cells in MOJ group was significantly lower compared with other two groups (**P<0.01). There was no significant difference between NJ and CON groups; CON, control; MOJ, malignant obstructive jaundice; NJ, nonjaundice; NS, not significant.

AB–PAS staining demonstrated that violetish goblet cells were evenly distributed and embedded in mucosa layer. However, the number of goblet cells and cellular component of goblet cells were decreased significantly in malignant obstructive jaundice group. Particularly, certain number of the goblet cells secreted considerable amounts of mucin into inner lumen. In addition, the mean optical density reflecting goblet cells density of malignant obstructive jaundice group (optical density, OD = 0.15 ± 0.02) was significantly lower than that of nonjaundice group (OD = 0.32 ± 0.04) and control group (OD = 0.35 ± 0.03, *P*<0.01). The amount of goblet cells had no significant difference between nonjaundice group and control group (*P*>0.05) (Figure 3).

#### Immunohistochemistry

**Figure 4:**
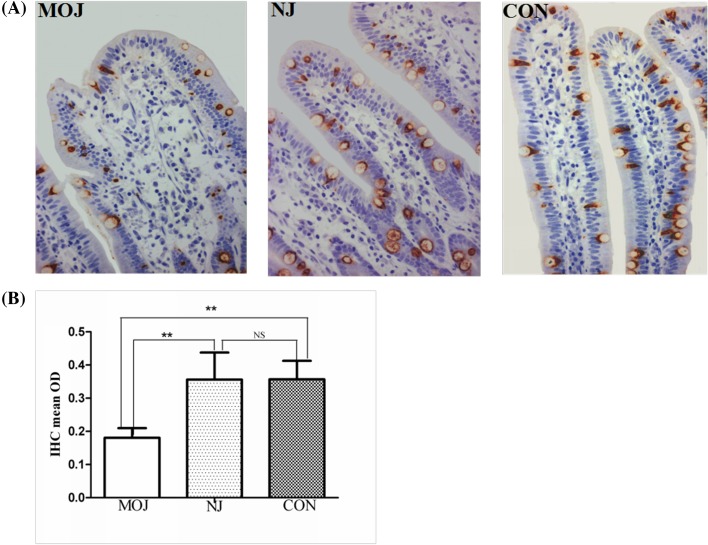
The expression of MUC2 in intestinal mucosa of patients from different groups (**A**) Immunohistochemistry. (**B**) The mean optical density of MUC2 in MOJ was significantly lower than the other two groups (**P<0.01). There was no significant difference between NJ and CON groups; CON, control; MOJ, malignant obstructive jaundice; NJ, nonjaundice; NS, not significant.

The immunohistochemical analysis showed that MUC2 was mainly in goblet cells. The amount of MUC2 in malignant obstructive jaundice group sections was decreased significantly compared with other two groups. The mean optical density of MUC2 was significantly lower (0.18 ± 0.03) in malignant obstructive jaundice group than that in nonjaundice group (0.33 ± 0.08) and controls (0.36 ± 0.08) (*P*<0.01). There was no significance difference between nonjaundice group and control group (*P*>0.05) (Figure 4).

#### Electron microscope

**Figure 5:**
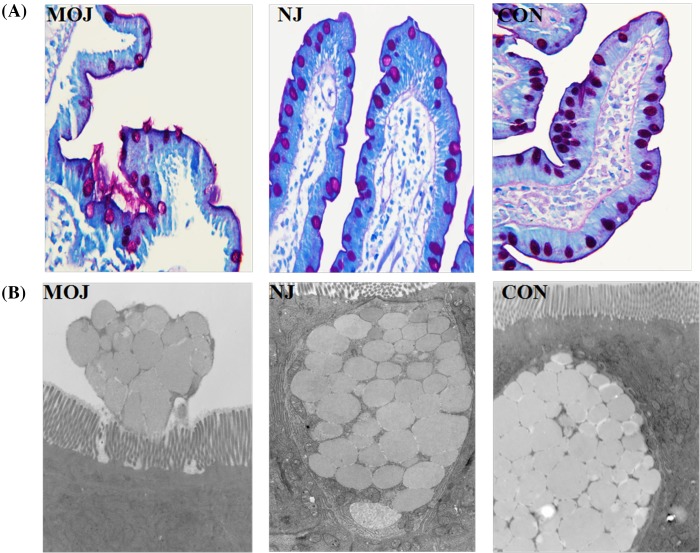
Secretion of goblet cells of small intestine from different groups of patients (**A**) AB–PAS staining. (**B**) Goblet cell under electron microscope. Goblet cells from MOJ group secrete enormous amount of mucoprotein. This phenomenon was not observed in goblet cells from NJ and CON group; CON, control; MOJ, malignant obstructive jaundice; NJ, nonjaundice.

The electron microscope demonstrated that mucosa villi displayed regular image in nonjaundice and control groups, and there are no obvious secretions from goblet cells. In contrast, villi from malignant obstructive jaundice group patients demonstrated short and disorder arrangement. Specifically, part of goblet cells can secret huge amount of mucin (Figure 5).

### Animal study

#### Morbidity and mortality of rats

There are five mortalities in total, two from obstructive jaundice group, one from internal biliary drainage group, and two from external biliary drainage group. Two were due to anesthesia overdose, the other three were due to biliary leakage. Ultimately, 35 rats were used for further experiments.

#### Biochemical tests in rats

**Figure 6:**
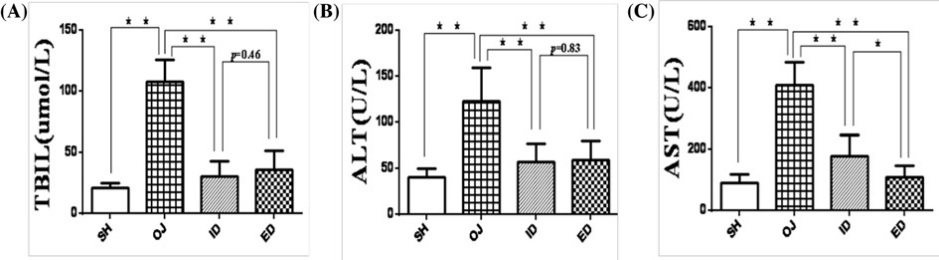
Liver function tests (TBIL, ALT, and AST) (**A**) TBIL was increased significantly in BDL rats compared with SH group (**P<0.01), either internal or external drainage significantly reduced TBIL. There was no significant difference between ID and ED groups (*P*=0.46). (**B** and **C**) Both ALT and AST were significantly increased in BDL rats compared with sham controls (**P<0.01), either internal or external drainage significantly reduced serum ALT and AST (**P<0.01). AST is significantly lower in ED rats compared with ID group. ALT was not significantly different between groups of internal or external drainage; ALT, alanine aminotransferase; AST, aspartate aminotransferase; ED, external biliary drainage; ID, internal biliary drainage; OJ, obstructive jaundice; SH, sham operation; TBIL, total bilirubin.

TBIL in obstructive jaundice group (107.72 ± 18.22 μmol/l) was significantly higher than that in sham group (21.46 ± 3.45 μmol/l, *P*<0.01). Internal biliary drainage (30.51 ± 12.44 μmol/l) and external biliary drainage (35.89 ± 15.57 μmol/l) decreased TBIL significantly (*P*<0.01). Whereas there was no significant (*P*>0.05) difference between internal biliary drainage and external biliary drainage groups (Figure 6A).

ALT (122.82 ± 36.23 U/l) and AST levels (409.48 ± 73.06 U/l) in obstructive jaundice group were significantly higher than those in sham rats (40.79 ± 9.24 and 88.72 ± 29.31 U/L, respectively, *P*<0.01). Internal biliary drainage and external biliary drainage significantly decreased ALT (56.79 ± 19.66 and 59.44 ± 20.11 U/l, *P*<0.01) and AST (178.49 ± 67.52 and 109.09 ± 38.06 U/l, *P*<0.01) levels. AST level in external biliary drainage decreased more compared with internal biliary drainage group (*P*<0.05). There was no significant difference in ALT level between external biliary drainage group and internal biliary drainage group (Figure 6B,C, *P*>0.05).

#### Hematoxylin–eosin staining

**Figure 7:**
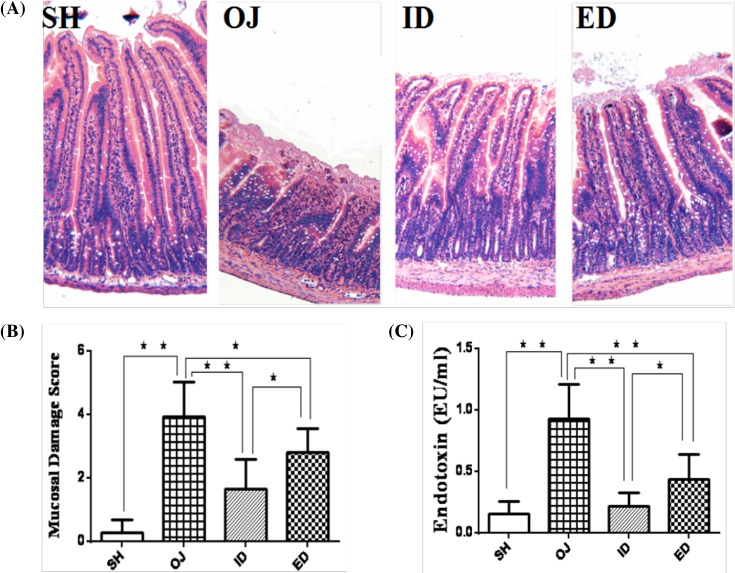
Small intestinal mucosa injury in rats of different groups (**A**) H&E stain. (**B**) The Chiu’s score was significantly increased in BDL rats compared with sham controls (**P<0.01). Both internal and external drainage significantly mitigated the Chiu’s scores. The effect of internal drainage was significantly better than external drainage. (**C**) Endotoxin levels of rats in different groups. Serum endotoxin was increased significantly in BDL rats compared with controls. The internal or external drainage significantly reduced endotoxemia. The effect of internal drainage was significantly better than external drainage; **P<0.01; *P<0.05; ED, external biliary drainage; ID, internal biliary drainage; OJ, obstructive jaundice; SH, sham operation.

H&E stain in sham group demonstrated that the intestinal villi were properly distributed, and mucosa, submucosa as well as other layers were clearly visualized under the microscope. There were subepithelial edema and crypts in internal biliary drainage and external biliary drainage groups without clear evidence of villous blunting. The intestinal tissue in obstructive jaundice group was damaged with villous blunting. The inner margin was enlarged and there were lamina propria lymphocytes and other inflammatory cells infiltration (Figure 7A). According to Chiu’s intestinal mucosal damage index, the score of rats with obstructive jaundice (3.93 ± 1.10) was significantly (*P*<0.01) higher than that of sham group (0.28 ± 0.40). The Chiu’s score of internal biliary drainage group (1.64 ± 0.94) and external biliary drainage group (2.81 ± 0.75) was significantly lower than that of obstructive jaundice group (*P*<0.01); internal biliary drainage group was significantly lower than external biliary drainage group (Figure 7B, *P*<0.05). The length of villi from obstructive jaundice group (175.14 ± 65.38 μm) was significantly shorter than that from sham group (457.67 ± 88.56 μm, *P*<0.01). The mucosa from internal biliary drainage and external biliary drainage group demonstrated improvement compared with obstructive jaundice group. Moreover, the serum endotoxin levels of different groups were corresponded to Chiu’s scores. The serum endotoxin level of obstructive jaundice group (0.93 ± 0.28 EU/ml) was significantly higher compared with that of sham controls (0.15 ± 0.10 EU/ml). Internal biliary drainage (0.21 ± 0.12 EU/ml) and external biliary drainage (0.44 ± 0.20 EU/ml) significantly decreased serum endotoxin level (*P*<0.01, compared with obstructive jaundice group); the internal biliary drainage has more efficacy than external biliary drainage (*P*<0.05) (Figure 7C).

#### AB–PAS staining

**Figure 8:**
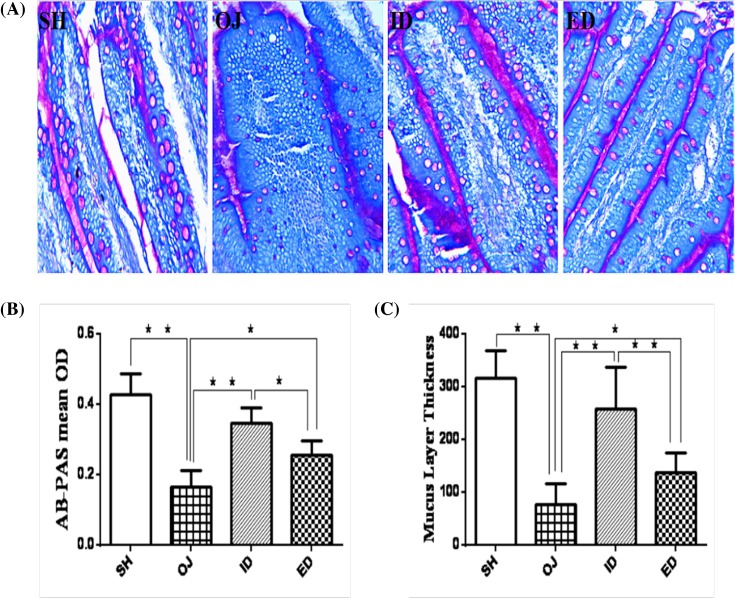
Changes of small intestinal goblet cells in rat of different groups (**A**) AB–PAS staining. (**B**) The OD was significantly reduced in BDL rats compared with sham controls. Internal or external drainage significantly increased the OD. The effect of internal drainage was significantly better than external drainage (**P<0.01; *P<0.05). (**C**) Small intestinal mucosa thickness of different groups. The mucosa thickness was significantly reduced in BDL rats compared with sham controls. The internal or external drainage significantly increased the mucosa thickness. The effect of internal drainage was significantly better than external drainage (**P<0.01; *P<0.05); ED, external biliary drainage; ID, internal biliary drainage; OD, optical density; OJ, obstructive jaundice; SH, sham operation.

AB–PAS stain in obstructive jaundice rats demonstrated the presentation of violet-reddish Goblet cells, the breakage of mucosa layer, leakage, and thinness of mucosa (Figure 8A). The mean OD of AB–PAS staining sections from obstructive jaundice group (0.16 ± 0.05) was significantly lower than that from sham animals (0.43 ± 0.06, *P*<0.01). The OD from internal biliary drainage (0.35 ± 0.05) or external biliary drainage (0.26 ± 0.04) groups was significantly higher than that from obstructive jaundice group (*P*<0.01). Moreover, internal biliary drainage is superior to external biliary drainage (*P*<0.05, Figure 8B). The mucosa layer of obstructive jaundice group was significantly thinner than that of sham group (77.14 ± 38.66 vs 316.5 ± 52.32 μm, *P*<0.01, Figure 8C). The internal biliary drainage (257.47 ± 80.57 μm) and external biliary drainage (137.38 ± 38.15 μm) significantly thickened the mucosa layer (*P*<0.01, Figure 8C). Moreover, the mucosa layer of internal biliary drainage group was thicker than that of external biliary drainage (*P*<0.05).

#### Immunohistochemistry assay

**Figure 9:**
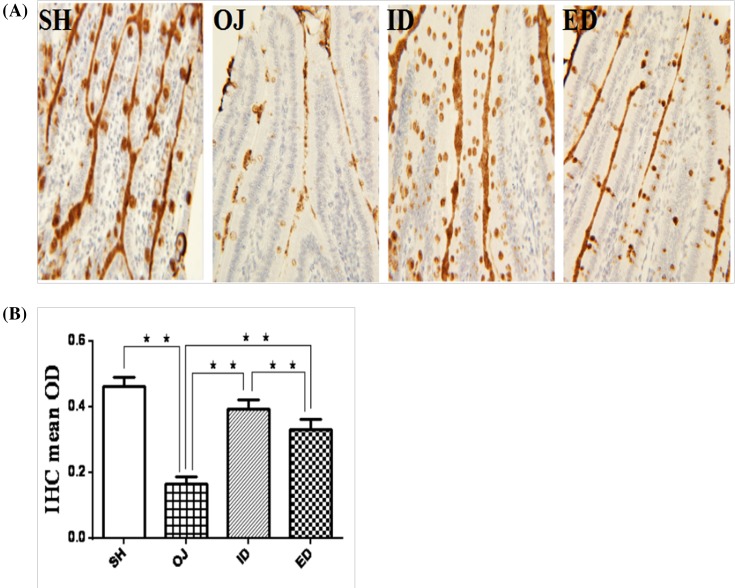
Expression of MUC2 in rat small intestinal mucosa of different groups (**A**) Immunohistochemistry MUC2 staining. (**B**) The OD was significantly reduced in BDL rats compared with sham controls; internal or external drainage significantly increased the OD. The effect of internal drainage was significantly better than external drainage (**P<0.01); ED, external biliary drainage; ID, internal biliary drainage; OD, optical density; OJ, obstructive jaundice; SH, sham operation.

The mucin-2 secretion in obstructive jaundice was significantly decreased in comparison with sham rats (0.16 ± 0.02 vs sham, 0.46 ± 0.03, *P*<0.01, Figure 9A). Internal biliary drainage (0.39 ± 0.03) or external biliary drainage (0.33 ± 0.03) significantly increased mucin-2 secretion compared with obstructive jaundice (*P*<0.01). Furthermore, internal biliary drainage was superior to external biliary drainage (*P*<0.05, Figure 9B).

#### Electron microscope

**Figure 10:**
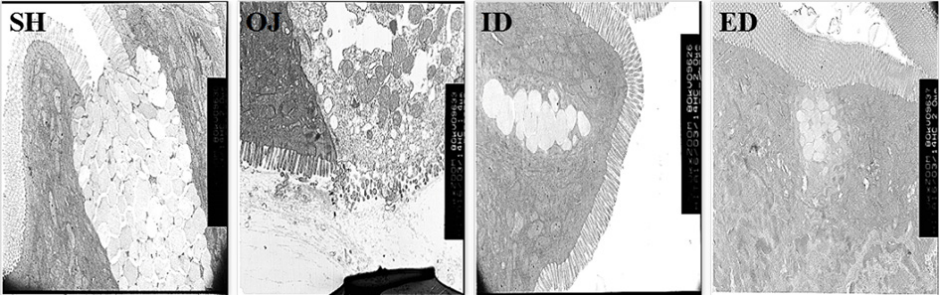
Secretion of small intestinal goblet cells in rats of different groups Electron microscope showed that goblet cells in OJ group secrete huge amount of mucoprotein. Goblet cells in the other three groups were plumped; ED, external biliary drainage; ID, internal biliary drainage; OJ, obstructive jaundice; SH, sham operation.

The intestinal villi from sham group had regular goblet cells which were fulfilled with small mucous granules. The intestinal villi from obstructive jaundice group demonstrated partially breakage, disorder arrangement, and sparsity characters. Part of goblet cells secreted mucus externally and vacuoles appeared within cells. In internal biliary drainage and external biliary drainage groups, mucosa villi displayed regular image, and there are no obvious secretions from goblet cells (Figure 10).

## Discussion

Previous research indicated that alternation of tight junction protein from intestinal epithelium and intestinal immune cell (T cell from intestinal epithelium) was the main cause of intestinal mucosal barrier damage [19–21]. However, no research about the intestinal epithelium goblet cells, which form the major line of defense, has been reported in patients with obstructive jaundice [22].

The present study investigated the patients with malignant obstructive jaundice and animals with bile duct ligation. We demonstrated that the reduction in small intestinal goblet cells resulted in the decrease in MUC2 secretion which played a major role in the damage of the intestinal mucosa. We also demonstrated that PBD attenuated mucosa damage, increased goblet cells, and reduced endotoxemia.

Significant reduction or even loss of bile acid in small intestine may possibly result in the reduction in MUC2 expression or goblet cells apoptosis. Several studies illustrated that bile acid up-regulates MUC2 expression. Pyo et al. [23] found that bile acid increases MUC2 expression in gastro cancer cells. In addition, Hu et al. [16] found that deoxycholic acid (one of the secondary bile acids) up-regulates the MUC2 gene expression in esophageal cancer cells. Also, Lee et al. [24] demonstrated that deoxycholic acid up-regulates MUC2 gene transcription in colorectal cancer cells.

Low pH related acidic environment within the intestinal lumen results in goblet cells reduction. Theodorou et al. [25] found that the status of goblet cells from Barrett’s esophagus endothelium was related to lumen pH environment. Particularly, bile acids stimulate goblet cell and goblet cell density in Barrett’s esophagus was increased compared with that of normal esophagus. This indicates that goblet cells are also influenced by pH and low pH environment is unsuitable for goblet cells growth. MUC2 is a NaHCO_3_-dependent cationic protein and decreasing pH increases the consumption of bicarbonate. However, Martin et al. [26] found that MUC2 positive goblet cells were significantly decreased when bile acid increased in ileum with necrotizing enterocolitis in early stage of life. The potential reason is immature ileum of infant.

Interaction between toxic components or bacteria and intestinal epithelium caused by MUC2 depletion leads to breakage of intestinal epithelium and cells shedding into the lumen [27], and as a result, the number of goblet cells is decreased because of shedding and cell apoptosis. The vicious cycle is, shedding of epithelial cells decreases MUC2.

The possible mechanism of the improvement of mucosa function induced by PBD is that liver function is improved, which improves the hematological function. The improvement of enteric blood circulation assists the growth of goblet cells and MUC2 synthesis. The potential mechanism that internal biliary drainage is superior to external biliary drainage is because internal biliary drainage restores the bile acid in hepato-enterological circulation which is close to human physical and anatomical environment. In addition, bile acid probably promotes the proliferation of goblet cells and expression of MUC2 because the bile alternatively alters the pH of intestinal lumen. Besides, the antioxidant effect of bile relieves the oxidative injury on intestinal mucosa [28].

Goblet cells from small intestinal villi secret huge amount of MUC2 through compound exocytosis [11], when obstructive jaundice occurs [29]. This may be caused by over growth of intestinal microbes and oxidative stress which induce the defense of Goblet cells. However, secretion of MUC2 accelerates goblet cell cytolysis, which significantly reduces the defendant capability [30,31].

As it is well known, gallstones related jaundice induces infections in patients. In order to exclude influence from the infection to MUC2 and goblet cells, we chose patients with malignant obstructive jaundice, healthy controls, and patients with malignant tumor without obstructive jaundice. Since we did not have clinical data about PBD, we established rat model of obstructive jaundice, external and internal biliary drainage. Our animal studies indicated that goblet cells and MUC2 were reduced in rats with obstructive jaundice, internal biliary drainage, and external biliary drainage increased goblet cells and MUC2. Our animal models mimic the clinical scenario.

It is well documented that bile acids increase goblet cells and MUC2, repair the damage of mucosa layer due to the lack of bile acids, and restore the intestinal mucosa barrier function. However, whether PBD benefits the patients remains debate. Eshuis et al. [2] found that there is no difference on survival time of patients with obstructive jaundice due to pancreatic head cancer between early surgery and delayed surgery because of PBD; van der Gaag et al. [3] also investigated the patients with obstructive jaundice due to pancreatic head cancer and found that PBD increases the complication rates. However, there is no significant difference on mortality and the length of hospital stay [32]. Chu et al. [4] found that effective biliary drainage improves the survival of patients with obstructive jaundice due to hepatocellular carcinoma. In a review article, Iskandar et al. [33] demonstrated that PBD significantly improves liver function, immune function, and nutritional status and prolongs patient survival. Although the documentations are not consistent, the majority of them are in favor of biliary drainage in patients with obstructive jaundice. In the present study, we investigated the mechanisms of biliary drainage on the improvement of patient outcomes.

## Conclusion

In conclusion, MUC2 and goblet cells demonstrated essential function of intestinal mucosa barrier protection. A possible mechanism of intestinal mucosa damage in patients with obstructive jaundice is the decrease in MUC2 and goblet cells. MUC2 and goblet cells of small intestinal increase significantly after biliary drainage, particularly in internal biliary drainage, which repairs the damaged mucosa layer and improves the barrier function.

## Abbreviations

AB–PAS: Alcian blue–periodic acid Schiff
ALT: alanine aminotransferase
AST: aspartate aminotransferase
BDL: bile duct ligation
CON: control
ED: external biliary drainage
H&E: hematoxylin–eosin
ID: internal biliary drainage
MOJ: malignant obstructive jaundice
MUC2: mucin-2
NJ: nonjaundice
OD: optical density
OJ: obstructive jaundice
PBD: preoperative biliary drainage
SH: sham operation
TBIL: total bilirubin
